# Understanding Panic Buying Through an Integrated Psychodynamic Lens

**DOI:** 10.3389/fpubh.2021.666715

**Published:** 2021-04-14

**Authors:** Marty A. Cooper, Jamie L. Gordon

**Affiliations:** SUNY Old Westbury, New York, NY, United States

**Keywords:** panic buying, COVID-19, psychological understanding, psychodynamic, interventions

## Abstract

Previous literature has identified panic buying as often being a response to environmental stressors. In early 2020, we saw an increase in panic buying as a response to a real and/or perceived lack of resources due to COVID-19. Although panic buying has a long history, there is a lack of literature to provide a psychological understanding of the phenomenon. During the early days of COVID-19 clients presented with fear and uncertainty. These negative emotions were, in part, a response to a real shortage of basic supplies. However, the panic response led to behaviors that, for some individuals, resulted in atypical buying patterns. From a therapeutic perspective, one can consider behavioral and psychodynamic explanations and interventions, and how this impacts the associated behaviors. This article will focus on psychodynamic understandings of panic buying as a response to events that result in negative emotions. By providing a psychodynamic understanding of panic buying, authors hope to contribute to the therapy of clients presenting with related behaviors and their associated negative affect.

In early 2020, we saw an increase in panic buying as a response to a real and/or perceived lack of resources due to COVID-19 (1–11). During the early days of COVID-19 clients presented with fear, panic, anxiety, and uncertainty (8). These negative emotions were, in part, a response to a real shortage of basic supplies. Within the availability of media coverage, there was a global witnessing of shortages of toilet paper, hand sanitizer, and groceries (4, 10). However, the panic response led to behaviors that, for some individuals, resulted in an increase in atypical buying patterns (1).

## Definition of Panic Buying

Previous literature has identified panic buying as often being a response to environmental stressors or during distressing and uncertain circumstances (12), including pandemics, war, governmental policy changes, or natural disasters (1, 3, 8, 11, 12). Panic buying has been defined as a behavioral phenomenon of a sudden increase in consumption and quantity of one or more necessary goods which is provoked by an adverse situation, which results in a disparity between supply and demand (1, 3, 4). In defining panic buying, it should be noted that the key difference in panic buying and other atypical consumer behaviors is the underlying motivation of the buying and the negative emotions that are associated with panic buying (8).

## Psychological Causes of Panic Buying

With the emergence of COVID-19 and the access to social media and information at our fingertips, panic buying has rapidly become a worldwide occurrence. With this worldwide occurrence brought the increase in research regarding panic buying; yet it remains under-researched (1, 9, 11), with a lack of empirical studies, which would further identify factors associated with panic buying (3) and different aspects of panic buying (1). Although panic buying has a long history, there is a lack of literature to provide a psychological understanding of the phenomenon (2). There is an absence of studies measuring the correlation between negative feelings and unpredictable events, which may lead to maladaptive purchasing behaviors (8, 11). Previous studies have highlighted key themes which are correlated with panic buying: uncertainty, fear and anxiety, a lack of trust, the perception of the crisis, social behaviors and conformity, a means of coping and a means of gaining control. Throughout times of crisis there is a heightened level of uncertainty that is experienced (3, 4, 7, 8, 10, 12). This includes uncertainty of when there would be an end to the crisis (2, 12) and uncertainty on whether or not there would be enough supplies to survive the duration of the crisis (7, 8, 12). Additional negative emotions that are correlated with panic buying include fear and anxiety, which are powerful drives in consumer behavior (3, 4, 8–10). This level of fear and anxiety are often associated with witnessing universal fear (12), the unknown (3, 11), and the future (8). Thirdly, negative emotions associated with this behavior include mistrust in the government during times of crisis, which may be impacted by previous governmental actions that were taken through times of crisis (2, 3, 10).

In evaluating panic buying, the individual's perception of the stressor is an important component. The perception of threat (2–4, 11), scarcity of goods (2–4, 11) risk (3), threat of losing control of the environment (2) or future and social demands (8), and feelings of insecurity and instability (13) all impact how the individual behaviorally reacts to the stressor.

The act of panic buying can symbolize several aspects in the individual. For instance, panic buying may be an attempt to conform to society (3, 4, 12). The act of mimicking witnessed behaviors is a means of herd conformity (1), measuring the intensity of the crisis by how others are reacting (7), and relying on social trust (11). Secondly, the act of panic buying been recognized as a coping and defense mechanism that individuals are engaging in (3, 7, 8, 11, 12). Through the use of panic buying, individuals are attempting to cope with the feelings of insecurity (2, 7) and to alleviate negative feelings (7). Lastly, the act of panic buying also symbolized a form of gaining control over a control-less situation (2–4, 7, 9, 12). By controlling the consumption patterns and available supplies, there is a decrease in the perceived lack of control.

According to Yuen et al. (11), the correlation associated with fear and increased purchasing can be further explained by one's mood congruency. The author's proposed that with negative emotions or heightened levels of stress, an individual's judgment to circumstances is negatively altered (11). Additionally, Jin et al. (7) found that the need to belong can influence how one copes with public health emergency, thus reducing panic buying. Working through interpersonal dynamics and systems with the individual allows for improvement in communication styles, correction of cognitive distortions relating to previous interactions, and an increase in traits including patience, empathy and tolerance (6). Further, the role of past experiences is associated with the patient's present behavior and defense mechanisms (6). Thus, if the individual is utilizing panic buying as a defense mechanism in order to create more adaptive coping mechanisms it is crucial for the clinician and the individual to process how the past is impacting the present.

Several sources have suggested that by providing adequate and consistent information on the situation, implementing purchase policies and governmental policies may prevent panic buying (1, 4, 10, 11). Notably, despite the understanding of the psychological background of panic buying that has been described throughout this article, there seems to be a lack of available guidelines and interventions for clinicians to utilize in therapy for clients presenting with these symptoms. This absence is highlighted by Arafat et al. (1), who noted that there is little evidence for the management of panic buying and that even though previous authors have proposed an online cognitive behavioral therapy (CBT) model for panic buying it lacks testing. In making therapeutic interventions available, there is a possibility of reduction in post-traumatic disorders following times of crisis, or to “combat the effects of pandemic-related fear” [8, p. 5]. Additionally, it has been noted to be difficult to breaking the cycle of panic buying (12), therefore interventions may reduce panic buying, while increasing adaptive coping mechanisms (10).

Adding to the psychological effects of COVID-19 comes the sudden shift in mental health treatment styles or availability, making the feasibility and practicality of treatment during a crisis an additional concern. Ingram and Best (6) acknowledged these shifts by discussing the impacts of psychiatric inpatient and outpatient facilities either closing or witnessing a lower census due to clients distancing from treatment centers; as well as the rapid shift from in-person therapy to tele-psychiatry. In considering the treatment of clients, it is crucial for clinicians to have an understanding of the impact that clinical restructuring to tele-psychiatry can have on both the clinician and the client.

Clinicians have verbalized concerns with tele-psychiatry due to the heavy reliance on non-verbal communication that it diminished through remote treatments (6). Clinicians have further demonstrated how tele-psychiatry is impactful in treatment through the example of help-rejecting clients (6). Reportedly, tele-psychiatry has exaggerated the act of help-rejecting clients rejecting efforts of reassurance provided (6).

In addition to the concerns of the clinical restructuring, many clinicians and clients have faced changes in the structuring of the sessions. Many clinicians have had to adjust their treatments toward practical considerations with client concerns being single- minded fear (6). With this single-minded fear, Ingram and Best (6) identified themes that have been discussed in therapeutic sessions including, the irony of feeling separate within a pandemic in which is being experienced globally, the new shift in the client's reality and the overwhelming sense of loss, mourning and isolation.

As previously stated, authors have proposed utilizing behavioral techniques and interventions in attempts to target specific groups who may be at risk of demonstrating, orr have a history of demonstrating behaviors associated with panic buying (10). With Arafat et al. (3) acknowledging the “erratic, irregular, episodic, sudden, unpredictable, and mostly happens during emergency situations (p.1)” nature of panic buying and the importance of practicing preventative measures (4), it provides the opportunity for clinicians to utilize behavioral interventions including safety planning for at risk clients. Along with behavioral therapy, clinicians can utilize the CBT intervention of distraction through exercise, non-pandemic related conversations, and shifting their attention away from the pandemic and material items (7), as well as by utilizing CBT skills including thought challenging and gathering evidence in order to reduce panic buying behavior (14). Further, studies have demonstrated that focusing on regret and worries is a prediction of panic buying; while focusing on the present moment, and the here-and- now is negatively correlated with panic buying (8). Thus, clinicians could incorporate Mindfulness Based Cognitive Therapy (MBCT) to educate the client on remaining in the present moment.

The term panic buying is compromised of the words “panic” and “buying;” which reflects both the affective and behavioral components of the occurrence (1). Therefore, though cognitive-behavioral and behavioral techniques can be useful, clinicians may consider using an integrated approach including a psychodynamic approach in therapy. In doing so, clinicians can use techniques to address cognitive distortions while further evaluating the negative affect of the individual and the impact of the shift in interpersonal relationships that may be being fueled by the distortions (14). Specific techniques that have been utilized by psychodynamic clinicians thus far have including “checking-in” at the commencement of sessions, being active rather than passive in session, universalize the situation in order to assist in normalizing their experience, provide validation of emotions and defense mechanisms, and be willing to disclose on your personal “new normal” (6). Additional techniques and interventions which have been utilized have included “crisis intervention therapy,” and “reality testing therapy” in order for the clinician to address the current, real needs of the individual (6).

## Psychodynamic Approach

Given the benefit of the research that has recently been produced to understand panic buying as a response to COVID−19, we can start to pair the findings of this research to areas of clinical inquiry for an integrated therapy approach. For settings that are not time limited in nature, this would allow the clinician to better understand how the client's history impacts their response to the pandemic and ultimately how it impacts panic buying behavior.

As previously stated, panic buying may be a form of coping and/or a defense mechanism (3, 7, 8, 11, 12). Considering this perspective, an integrative approach may work to understand the client's developmental history and historic use of coping strategies and defense mechanisms. For instance, does the client have a history of intellectualization or focusing on the intellectual part of something to avoid the emotions associated with it? Does the client engage in “acting out” behaviors? Our ability to understand the client's patterns may help us to understand the underlying coping mechanism or defense of the panic buying behavior.

In addition to understanding the coping or defense mechanisms, we can utilize the knowledge of the developmental history of the client to understand what negative experiences they had and which of the associated emotions or cognitions are similar to what they are experiencing during the pandemic. Research has indicated that uncertainty, fear, anxiety, lack of trust, perception of the crisis, social behaviors, conformity, a means of coping or gaining control, and uncertainty (3, 4, 7, 8, 10, 12), threat of losing control of the environment (2) or future and social demands (8), and feeling of insecurity and instability (15) are all associated with panic buying. This provides us important guidance as we identify these themes in our client's history including negative affect, unsafe environments, lack of resources, etc. This will help us to understand if our clients may be vulnerable to similar negative affect which may result in the coping behaviors or defense mechanisms associated with panic buying. In addition to understanding the affective history of our clients we may also want to consider systemic oppression. Research suggests that mistrust in government and political measures are associated with panic buying (2, 3). Some clients will have experienced systemic oppression or belong to groups that have been historically mistreated by government and/or disproportionately negatively impacted by politics. We may also be mindful of how our own industry may have inappropriately treated individuals and how this may impact their engagement in the therapeutic process.

**Figure 1:**
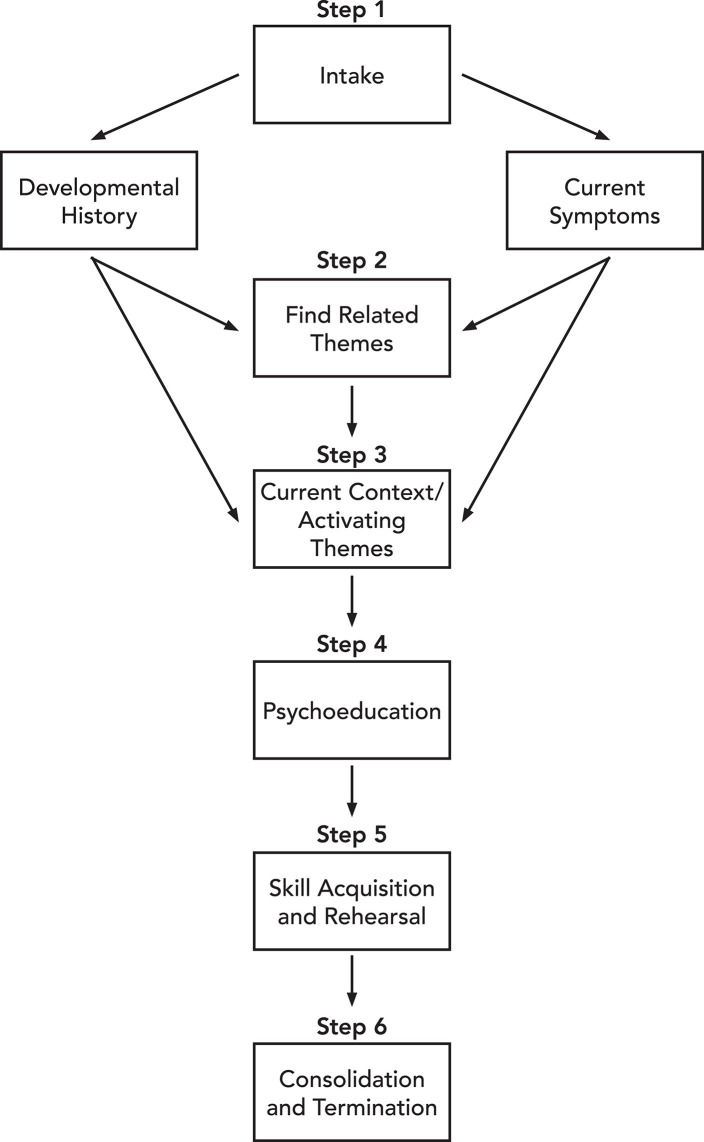
Integrated psychodynamic model.

Figure 1 provides a visual representation of the integrated treatment model. Step 1 Intake, is where we collect the current symptoms, including physiological reactions, as well as the developmental history. In Step 2 Finding Related Themes, we determine the related themes between the developmental history and current symptoms. Then, in Step 3, Current Context/Activating Themes, we work with the client to understand how and when the current context, COVID 19, is activating themes from their developmental history and what associated physiological symptoms manifest at these times. Step 4, Psychoeducation, provides psychoeducation including mindfulness training to help manage the symptoms and ameliorate any physiological manifestation. In Step 5, Skill Acquisition and Rehearsal, we assign the skills as homework and ask the client to record progress until amelioration of symptoms is achieved to the point that the client can end treatment. Finally, Step 6, Consolidation and Termination, is an opportunity to provide consolidation of the work that has been done.

## Limitations

As with all therapeutic approaches there are limitations. First, clients may not approach therapists for the treatment of panic buying. Second, the condition may not require treatment as prevention can be carried out by alternate public measures. Third, this psychodynamic approach may take more time and be prohibitive for some clients or within some clinical settings.

## Conclusion

Important work is being done to understand panic buying and provide treatment models to assist clients when panic buying surfaces in therapy settings. Recent literature has highlighted the experience of individuals engaging in panic buying. Additionally, we have gained therapy models like a CBT model that may provide much needed relief for clients during this time of stress.

In addition to the rapidly developing literature, this article has discussed that working through the client's developmental history may help the client better understand their reaction to the pandemic and the associated behaviors they engage in. This knowledge may facilitate therapy and encourage their engagement in the tools or skills the therapist can integrate into the therapeutic plan based on the model provided. Furthermore, this may better prepare them for future unpredictable events. Utilizing an integrated therapy approach may provide the client with an increased understanding of themselves while still providing the practical components necessary to address panic buying behaviors.

## Data Availability Statement

The original contributions presented in the study are included in the article/Supplementary Material, further inquiries can be directed to the corresponding author/s.

## Author's Note

MC Department of Psychology, Mental Health Counseling Program, State University of New York (SUNY) College at Old Westbury. JG South Oaks Hospital, Northwell Health Long Island, United States.

## Author Contributions

MC and JG contributed to concept and development of the article and wrote the first draft of the manuscript. All authors contributed to manuscript revision, read, and approved the submitted version.

## Conflict of Interest

The authors declare that the research was conducted in the absence of any commercial or financial relationships that could be construed as a potential conflict of interest.
